# Why do Asian-American women have lower rates of breast conserving surgery: results of a survey regarding physician perceptions

**DOI:** 10.1186/1471-2458-9-246

**Published:** 2009-07-17

**Authors:** Jane T Pham, Laura J Allen, Scarlett L Gomez

**Affiliations:** 1 Northern California Cancer Center, Fremont, California (JTP, LJA, SLG), USA; 2 Division of Epidemiology, Department of Health Research and Policy, Stanford University School of Medicine, Stanford, California (SLG), USA

## Abstract

**Background:**

US Asian women with early-stage breast cancer are more likely to receive a modified radical mastectomy (MRM) than White women, contrary to clinical recommendations regarding breast conserving treatment (BCT).

**Methods:**

We surveyed physicians regarding treatment decision-making for early-stage breast cancer, particularly as it applies to Asian patients. Physicians were identified through the population-based Greater Bay Area Cancer Registry. Eighty (of 147) physicians completed a questionnaire on sociodemographics, professional training, clinical practices, and perspectives on the treatment decision-making processes.

**Results:**

The most important factors identified by physicians in the BCT/MRM decision were clinical in nature, including presence of multifocal disease (86% identified this as being an important factor for selecting MRM), tumor size (71% for MRM, 78% for BCT), cosmetic result (74% for BCT), and breast size (50% for MRM, 55% for BCT). The most important reasons cited for the Asian treatment patterns were patient attitudes toward not needing to preserve the breast (53%), smaller breast sizes (25%), and fear and cultural beliefs (12%).

**Conclusion:**

These survey results suggest that physicians perceive major roles of both clinical and cultural factors in the BCT/MRM decision, but cultural factors may be more relevant in explaining surgical treatment patterns among Asians.

## Background

For most patients with early-stage breast cancer, breast conserving treatment (BCT) provides a less invasive alternative to modified radical mastectomy (MRM) with equivalent survival [1] and possibly also better quality of life [2,3]. The use of BCT, however, occurs disproportionately in the US, with substantial ethnic, geographic and socioeconomic variation [4-8]. Asian women have MRM at significantly higher rates [5,7,9-11]; for example, in California, among stage 0–II breast cancer patients diagnosed in 1988–1995, 67.5% of Asians had MRM compared to 57.3% of Whites [5]. This proportion is even higher among certain Asian populations; more current data from the national Surveillance, Epidemiology, and End Results (SEER) program show that 54% of Vietnamese, 53% of Filipinas, and 48% of Chinese diagnosed with early-stage breast cancer between 1988–2005 had MRM, compared to 39–42% of non-Hispanic Whites, Blacks, and Hispanics [9,12]. BCT, which typically requires an average of five weeks of adjuvant radiation, may be less feasible for those who live far from treatment facilities and/or have travel limitations [13,17]. There are also clinical contraindications to BCT, such as small breast size, and a high tumor to breast size ratio [1], although it is unclear whether these clinical contraindications would explain the substantially lower rates of BCT among certain Asian populations. Other factors such as poor patient-provider communication, language barriers, and cultural factors may also affect the patterns seen in cancer surveillance data, yet patient sociodemographic or tumor characteristics have not been shown to explain the differences in treatment patterns across ethnic groups [7,11,18]. Qualitative research suggests that Asian cultural characteristics and beliefs about physical appearance, fate, and the safety and effectiveness of BCT, as well as difficulties with adjuvant treatment and cross-cultural misunderstandings in the doctor-patient encounter, may explain the higher frequency of MRM over BCT [10,19].

Physician perspectives can provide valuable insight into the decision-making process, and consequently, may help to identify reasons for racial/ethnic variations in treatment patterns. Factors such as provider gender [20], period of provider training [21], extent of communication between physician and patient [20], and hospital of diagnosis or distance to hospital or treatment facility [22] appeared to be associated with differences in choice of MRM between Black and White patients. The literature also documents limited concordance between patients' preferred role, with regards to autonomy and participation, and physician perception of this role in the decision-making process for breast cancer treatment [23], and for cancer treatment in general [24].

Because of the documented importance of physicians' perspectives in decisions regarding cancer treatment, and for understanding the persistently higher rates of MRM among Asian populations, we surveyed physicians from the Greater San Francisco Bay Area on their perspectives regarding the factors important in deciding between BCT and MRM. This survey is part of a larger pilot study of decision-making processes as they relate to ethnic disparities in receipt of optimal breast cancer treatment. This pilot study also included focus groups with breast cancer patients, and qualitative one-on-one interviews with patients and a "co-decision maker," and was designed to be a mixed methods study that incorporated all of the information gathered from the patient, co-decision maker, and provider interviews into a quantitative instrument, which was subsequently cognitive and pilot tested.

## Methods

### Selection of Physicians

Physicians were identified through the nine-county, population-based Greater Bay Area Cancer Registry (GBACR), a participant in SEER and in the California Cancer Registry (CCR). The GABCR covers a region of approximately 3 million residents and a racially/ethnically, socioeconomically, and geographically diverse population [25]. The names of the attending and follow-up physician of each breast cancer case, as well as their mailing address, are available from GBACR data. Other data, including physician sociodemographics and clinical specialty, are not available. Based on GBACR data for approximately 700 Chinese, 500 Filipina, and 120 Vietnamese incident breast cancer patients diagnosed during the years 2002–2004, we generated an eligibility sample of 50 physicians who had diagnosed and/or treated proportionally the most patients in each of these three racial/ethnic groups, for a total eligibility sample of 150 physicians.

### Survey Instrument

The pre-tested, self-administered, mailed questionnaire consisted of 21 questions on physician socio-demographics, professional training and specialty, reported practices and guidelines, and perspectives on the treatment decision-making processes and factors considered important in treatment decisions. We also inquired about the overall racial/ethnic composition of each physician's patients, the extent to which clinical practice guidelines (or equivalent) were employed, and the estimated proportion of early-stage breast cancer patients treated with MRM. Questions about treatment decision-making processes developed by Bruera et al. [24] and Degner et al. [26] were adapted for use in this study. We also elicited physicians' perspectives on reasons why they thought Asian patients diagnosed with early-stage breast cancer are more likely to have MRM over BCT compared to the general population.

### Data Collection

Eligible physicians were mailed, in 2005, a packet that included a cover letter describing the study and inviting them to participate by completing the enclosed questionnaire; completed questionnaires were received in 2005–2006. As an incentive to return the survey, a money order for $15 was included. Telephone calls were made to encourage participation. Of the 150 physicians, three were deemed ineligible, and 80 surveys were completed, yielding a response rate of 54%. Since the physicians were identified through the cancer registry, information on sociodemographic or other characteristics such as specialty type were not available for assessing whether respondents were different from non-respondents.

### Statistical Analysis

The primary outcomes of interest were information about physician practices and perspectives regarding treatment decision-making. We conducted descriptive analyses of the physicians' reported practices and perspectives (Tables 1 and 2). We also conducted exploratory multivariable logistic regression of the associations between selected physicians' practices and perspectives and physician demographic characteristics. Analyses were performed using the R statistical program [27].

**Table 1:** Distribution of patient characteristics, practices, and guidelines, as reported by physicians (N = 80), Greater San Francisco Bay Area, 2005–2006

*Characteristics, practices, guidelines*	*N*	None	A Few (<20%)	Several	Most (>90%)
		
		% *	% *	% *	% *
Race/Ethnicity of patients treated over last 2 years					
Non-Hispanic White	75	1.3	9.3	60.0	29.3
Hispanic	76	6.6	31.6	60.5	1.3
African American/Black	77	9.1	52.0	39.0	0.0
Asian/Pacific Islander	78	1.3	21.8	65.4	11.5

Patient decision-making preferences for treatment					
Patient alone	76	18.4	51.3	17.1	13.2
Patient with physician input	78	0.0	10.3	39.7	50.0
Shared decision	78	0.0	15.4	38.5	46.2
Physician with patient input	76	13.2	60.5	21.0	5.3
Physician alone	77	45.4	42.9	11.7	0.0

					
		
Hospital has facility-specific clinical practice guidelines (CPG) for patients		Yes		No	
		
	80	27.5		72.5	

Number of patients for whom physicians employ these CPG (among practices with CPGs, N = 23)	23	0.0	8.7	4.4	87.0

Number of stage I and II breast cancer patients treated with MRM	74	1.4	68.9	25.7	4.0

*Missing responses not included in percent calculations.

**Table 2:** Physician perspective on factors determining treatment decisions (N = 80), Greater San Francisco Bay Area, 2005–2006

*Physician perspective*	% indicating "yes" *
Most important factors for stage I and II patients treated with MRM over last 2 years	
Presence of multifocal or multicentric disease	86.2
Tumor size	71.2
Patient's attitude toward preserving the breast	66.2
Breast size	50.0
Age	28.8
Location of tumor	25.0
Patient's ability/willingness to travel to receive adjuvant radiation therapy	22.5
Family history	18.8
Cosmetic result	17.5
Adverse effects/outcome	6.2

Most important factors for stage I and II patients treated with BCT over last 2 years	
Patient's attitude toward preserving the breast	87.5
Tumor size	77.5
Cosmetic result	73.8
Breast size	55.0
Patient's ability/willingness to travel to receive adjuvant radiation therapy	38.8
Presence of multifocal or multicentric disease	32.5
Age	30.0
Location of tumor	22.5
Adverse effects/outcome	7.5
Family history	6.2

Reasons why Asian subgroups are more likely to get MRM over BCT	
Patient's attitude toward preserving the breast	52.5
Breast size	25.0
Age	11.2
Patient's ability/willingness to travel to receive adjuvant radiation therapy	10.0
Tumor size	8.8
Adverse effects/outcome	8.8
Cosmetic result	7.5
Presence of multifocal or multicentric disease	6.2
Location of tumor	5.0
Family history	2.5
Don't know	17.5
Other	18.8
Cultural factors	6.2
Fear	6.2

*Non-response was counted as "No" for that factor.

## Results

Table 3 shows the frequencies of physician demographic characteristics. A relatively large proportion of our respondents were male (66%), ranged in age from 40 to 59 years (65%), and self-identified as non-Hispanic Whites (63%). Most of the physicians were US-born (73%), over half spoke English only (59%), but many have had to discuss treatment in a foreign language (64%, often through an interpreter). 18% percent of physicians received training in oncology during their residency, and 35% during their fellowship.

**Table 3:** Distribution of physician demographic characteristics (N = 80), Greater San Francisco Bay Area, 2005–2006

*Characteristic*	*n*	% *
Gender		
Male	53	66.2
Female	27	33.8
Age (years)		
Under 40	11	13.8
40–49	22	27.5
50–59	30	37.5
60 and over	17	21.2
Race/Ethnicity		
Asian	26	32.5
Non-Asian	54	67.5
Country of Origin		
USA	58	72.5
Not USA	22	27.5
Age Immigrated to US		
US Born	58	72.5
<18 yrs old	10	12.5
18+ yrs old	12	15.0
Foreign Language Fluency		
English only	47	58.8
Speaks more than English	33	41.2
Discussed treatment in foreign language		
Yes	51	63.8
No	23	28.8
Residency specialty		
Medicine/Family	17	21.2
General Surgery	48	60.0
Radiation Oncology	14	17.5
Fellowship specialty		
Breast or Oncology	28	35.0
Other specialty	13	16.2
None	39	48.8

*Missing responses not included in percent calculations.

Physician practices and policies are reported in Table 3. Largely attributable to our selection criteria, 77% of physicians reported that "several" (20–90%) to "most" (greater than 90%) of the breast cancer patients they treated over the past two years were Asian. Only 4% treated more than 90% of their stage I and II breast cancer patients with MRM. In general, the physician-reported proportions of patients receiving MRM appear to be slightly lower than those based on population-based cancer registry data. These differences may be due to several factors, including some recall bias on the part of the physicians, and the differences in their patient populations by race/ethnicity and other sociodemographic factors.

Physician perspectives on important factors in the treatment decision are shown in Table 2. Frequently reported factors include clinical factors (tumor size, multifocal disease), breast size, and patient's attitude toward preserving the breast. For patients treated with BCT, 87.5% of physicians considered patient's attitude toward preserving the breast to be the most important factor, and 74% of physicians also considered cosmetic result to be important.

53% of physicians believed Asian women are more likely to have MRM because of patient attitudes about breast preservation, and 25% believed that breast size also plays a strong role. Physicians also listed fear and/or cultural beliefs as "other factors" influencing MRM decisions among Asian women.

We assessed associations between physicians' practices and perspectives and physician demographic characteristics using logistic regression (data not shown). Although based on a small sample, we found that Asians were less likely than physicians of other ethnicities to report cosmetic result as an important factor for patients choosing BCT (OR = 0.13, 95% CI = 0.04–0.40); male physicians were more likely than females to think cosmetic result was important in BCT decisions (OR = 5.22, 95% CI = 1.76–15.49), and less likely to perceive that patient travel concerns were important (OR = 0.21, 95% CI = 0.08–0.58). When attempting to explain Asian treatment patterns, Asian physicians were more likely to perceive that patient age (OR = 5.00, 95% CI = 1.10–22.63) and travel concerns (OR = 7.65, 95% CI = 1.38–42.56) were important factors. The full logistic regression results are available upon request.

## Discussion

Breast cancer is the most commonly diagnosed cancer among Asian women [28-30], with the majority of cancers diagnosed at an early-stage. Because BCT is a recommended alternative to MRM, the fact that the vast majority of Asian women still have MRM is a concern, particularly if this treatment pattern reflects disparities in the delivery of guideline-recommended treatment. We found that physicians believed that patient attitudes toward [not] preserving the breast was the predominant reason for the higher rate of MRM among Asian women compared to other racial/ethnic groups. This finding supports suggestions from qualitative studies that a primary reason for the observed surgical treatment pattern among Asians is reduced emphasis on physical appearance when making treatment decisions, reflective of cultural norms that place less significance on the breasts in particular [10,19]. This physician survey was conducted as part of a larger pilot study to evaluate such cultural factors and the role that they play in the complex decision-making process among Asian women diagnosed with early-stage breast cancer.

The second most commonly cited reason for the Asian surgical treatment pattern was breast size, given that BCT is often not clinically feasible considering the relatively small breasts among Asian women [1]. Our study did not show tumor size to be considered an important reason, which is consistent with prior studies that either have not shown a difference in tumor size distribution between Asian and non-Asian women [7,18] or modest differences that were insufficient to explain the ethnic treatment differences [8,9]. These findings together suggest that MRM is more often performed on Asian women possibly because of contraindications in large tumor-to-breast ratios, which is primarily driven by small breast size, rather than large tumor sizes; however, tumor-to-breast ratio alone seems unlikely to explain all of the ethnic treatment differences. Because our findings consist of physician beliefs, further studies based on objective measures are needed to verify this finding.

The role of patient-provider cultural concordance may be relevant when examining Asian physicians' perspectives on decision-making process and treatment factors. Do Asian physicians draw from their own cultural experiences when interacting with Asian patients and when recalling patient treatment decisions? For example, difficulty in travel to receive adjuvant therapy (required as part of the full course of BCT) has been often considered as a reason for receipt of MRM over BCT [13-17], and proposed as a reason for the Asian treatment pattern [7]. Although few doctors in our study believed that this would explain why Asian patients are less likely to choose BCT, six of the eight doctors in our study that did perceive travel concerns to be a reason for the Asian treatment difference were of Asian race/ethnicity. Asian physicians were also much more likely to report that patient age may explain why Asian patients are more likely to choose MRM. It is difficult to determine whether this suggests that they believe that Asian breast cancer patients have a different age distribution from non-Asian patients, or whether they believe that Asian patients strongly link age to other treatment-relevant factors such as cosmetic result, attitude toward preserving the breast, and ability to travel. Asian physicians were also more likely to report that patients prefer that the physician makes the decision alone, suggesting cultural concordance as well as an implicit acknowledgement of the cultural belief in deference over medical professionals. Cultural concordance may play positive roles in patient care, as it has been associated with greater satisfaction with care [31], use of needed health services and less delay in seeking care, although these associations have been shown to be stronger for Whites and Blacks and less marked for Hispanics and Asians [32]. However, race concordance may result in poorer care; for example, it has been observed that physician sympathy for patient beliefs regarding cultural modesty and lack of emphasis on the importance of preventive care was associated with fewer recommendations for breast and cervical cancer screening [33].

The design aspect of this survey was limited in its ability to explore other factors in the decision-making process and possible explanations for Asian treatment patterns. For example, the study did not match providers to patients and did not attempt to examine concordance between patient and physician responses. Additionally, because physicians were selected based on the proportion of Chinese, Vietnamese, or Filipina patients treated, and given the heterogeneity among Asian subgroups, these results may not be applicable to patients of other Asian ethnicities.

Although this physician survey was conducted as part of a larger pilot study and was designed to be exploratory in nature, we were able to show that, from physicians' perspectives, while the main factors in deciding between BCT and MRM are clinical in nature, major reasons for the observed differences in surgical treatment patterns among Asian women are also believed to be psychological and cultural in nature. A main reason cited for electing BCT in general, and for the Asian treatment pattern is attitude toward preserving the breast. Although many of these cultural factors require additional research for a more complete understanding of their role and significance, physician awareness of these factors will allow them to incorporate this knowledge into their interactions with Asian breast cancer patients. In cases where there are no clear clinical contraindications to BCT, physicians should directly address the patient's attitude toward preserving the breast as well as other cultural factors. When a patient expresses that preserving the breast is not important, are they actually masking a fear of adverse outcomes or adjuvant therapy? Providers should be sure that patients are fully informed of their treatment options and of the consequences of each option. Finally, if travel is a barrier, as suggested through other studies of the general population [13-17], it should be possible to remove this barrier, either by providing private travel services or through coordination with existing public health or community programs. Through deeper understanding of the treatment decision-making process among Asian women is needed, early efforts such as these could contribute to reducing the racial/ethnic disparities in receipt of optimal breast cancer treatment.

## Conclusion

In conclusion, this study of providers who have treated early-stage Asian breast cancer patients in the Greater San Francisco Bay Area showed that physicians perceive major roles of both clinical and cultural factors in the breast conserving therapy versus modified radical mastectomy decision, but psychological and cultural factors may be more relevant in explaining the previously documented higher MRM patterns among Asians.

## Authors' contributions

All of the authors designed and implemented the study, and contributed to the writing of the manuscript. JTP took the lead in conducting the analysis and writing the manuscript. All authors have read and approved the final manuscript.

## Pre-publication history

The pre-publication history for this paper can be accessed here:


